# Supplementary material for: Political prioritization and the competing definitions of adolescent pregnancy in Kenya: An application of the Public Arenas Model
Source: PLoS One. 2020 Sep 14;15(9):e0238136. doi: 10.1371/journal.pone.0238136 (PMC7489501; doi:10.1371/journal.pone.0238136)
Supplement: S1 File — (DOC) [file pone.0238136.s001.doc]

**S1 File: Consolidated criteria for reporting qualitative research (COREQ) checklist**

Tong A. Sainsbury P. Craig J. Consolidated criteria for reporting qualitative research (COREQ): a 32-item checklist for interviews and focus groups. International Journal for Quality in Health Care. 19(6): 349-357

| **Item** | **Response** | **Location in manuscript (section, page number)** |
| --- | --- | --- |
| ***Domain 1: Research team and reflexivity*** | | |
| *Personal Characteristics* | | |
| 1. Interviewer/ facilitator | Experienced interviewers/moderators | Data management and analysis, page 5 |
| 2. Credentials | MAO holds MBChB, MSc, PHD; GR holds an MD and MPH, JSW holds a PHD, EG has an MD EB is MBChB, MMeD, MPH, PhD, MBE, CIP; CB has a DrPH, | Title Page |
| 3. Occupation | MO is a medical doctor and Global Health Research Scientist, GR is a professor in epidemiology, Program Coordinator, EB is Chief Research Officer & Co-Director of Research Care Training Program; JSW is an Assistant Professor, Institute for Health Policy Studies at UCSF; CB is a professor of paediatrics and obstetrics at UCSF, EG is the director of the UCSF Centre for Implementations sciences | Information on author occupation is not available in the manuscript, only affiliations available |
| 4. Gender | MO, EB and CB are females, GR, JW, and EG are males | Not in the manuscript |
| 5. Experience and training | All authors are technically and methodologically experienced researchers in Kenyan settings | Data management and analysis page 5, Title Page |
| *Relationship with participants* | | |
| 6. Relationship established | Some participants might have had previous working relationship with MO and EB through interactions in technical working groups | Recruitment, pagse 4&5 |
| 7. Participant knowledge of the interviewer | Participants were informed about the reasons for the research via the invitation to participate and the consent form. | Recruitment, pages 4&5 |
| 8. Interviewer characteristics | No interviewer-related biases identified. |  |
| ***Domain 2: Study design*** | | |
| *Theoretical framework* | | |
| 9. Methodological orientation and theory | Thematic analysis approach. The coding framework based on literature, topics from interview guides, and emerging themes from transcripts. | Data management and analysis, page 6&7 |
| *Participant Selection* | | |
| 10.Sampling | Participants were purposively selected for maximum variation in representation across different sectors. | Recruitment: pages 4&5 |
| 11. Method of approach | The lead researcher and a representative from the Ministry of health, division of reproductive and maternal health identified potential participants. Participants were then contacted via phone call and given a brief overview of the study and asked if they were willing to participate. | Recruitment: Methods, pages 4&5 |
| 12. Sample size | 14 National state and non-state actors from government, civil society, donor organisations, religious society, youth and education sector, health officials | Recruitment, pages 4&5 |
| 13. Non-participation | All eligible and approached individuals agreed to participate in the study. | N/A |
| *Setting* | | |
| 14. Setting of data collection | Interviews were conducted in private setting at the participants choice of place | Data management and analysis, page 6&7 |
| 15. Presence of non-participants | No | N/A |
| 16.Description of sample | National state and non-state actors, > 18 years of age involved in adolescent health policy making  All participants provided their consent to participate in the study. | Recruitment; page 4. Ethical consideration and protection of human subjects, page 5-6, |
| *Data collection* | | |
| 17. Interview guide | The in-depth interview guide used in the study is included in S2 file and included questions on 1) the current state of priority for adolescent SRH in the health agenda of Kenya, 2) who holds significant influence on how adolescents and adolescent SRH are defined and addressed, and 3) how adolescent SRH can be re-framed to political leaders in order to generate political support and bring it to the agenda table. | Data collection page 5-6; Ethics approval and consent to participate, page 5-6 |
| 18. Repeat interviews | N/A | N/A |
| 19. Audio/ visual recording | All interviews were audio recorded. Audio-recordings were transcribed, translated and coded using Dedoose software. Interviews were digitally recorded and transcribed verbatim by professional transcriptionists, excluding any identifying information. All files were password-protected and stored in a secure location. | Data collection, page 5 |
| 20. Field notes | Interviewers took notes for purposes of assistance with transcription. | Data collection. Page4-5 |
| 21. Duration | Each interview lasted approximately 90 minutes | Data collection, page 4-5 |
| 22. Data saturation | Data saturation was discussed and it was concluded that the data saturation was reached. | Recruitment , page 4 |
| 23. Transcripts returned | N/A | N/A |
| ***Domain 3: Analysis and findings*** | | |
| *Data analysis* | | |
| 24. Number of data coders | A team of two researchers coded the transcripts. Consistency of coding between two ndividuals was established by initially coding the same transcripts and through frequent discussion between coders until consistency was fully established. | Data management and analysis page 5 |
| 25. Description of coding tree | Thematic analysis approach was utilized. The coding framework based on Public Arenas Model literature, topics from interview guides, and emerging themes from transcripts. | Methods, page 5 |
| 26. Deviation of themes | The coding framework based on the Public Arenas Model as well as literature, topics from interview guides, and emerging themes from transcripts. | Methods, page 5; Table 1, page 20 |
| 27. Software | Dedoose qualitative software program (Sociocultural Research Consultants, LLC) was utilized. | Data collection, page 5 |
| 28. Participant checking | Findings are not yet disseminated |  |
| *Reporting* |  |  |
| 29. Quotations presented | Participant quotations are provided to illustrate the themes and attributed to participants. De-identified participant characteristics are provided for each quote to distinguished between type gender and state and non state actors | Results, pages – 6-13 |
| 30. Data and findings consistent | N/A | N/A |
| 31. Clarity of major themes | Four major themes are clearly presented with rich narratives | Results, pages– 6-13 |
| 32. Clarity of minor themes | Minor themes were in line with the Public Arenas Model conceptual framework are discussed | Results, pages– 6-12 |
