# Supplementary material for: Political prioritization and the competing definitions of adolescent pregnancy in Kenya: An application of the Public Arenas Model
Source: PLoS One. 2020 Sep 14;15(9):e0238136. doi: 10.1371/journal.pone.0238136 (PMC7489501; doi:10.1371/journal.pone.0238136)
Supplement: Attachment — Submitted filename: Review comments- Political prioritization and the competing definitions of adolescent pregnancy in Kenya manuscript.docx [file pone.0238136.s003.docx]

This paper is very important for a country where sexual reproductive health and rights, especially teenage pregnancies and verses social development goals are of great concern in public and political domains. While the paper needs a strong argument, it is a great contribution to current SRHR bill debates.

I have read this paper with great pleasure. I feel it has great potential. The paper provides a substantial contribution SRHR bill and policy development practices in Kenya, and where such debates on teenage pregnancies have brought about sharp divisions between conservatives-the opposition- and liberals -those promoting rethinking policy environments in the light of SRHR.

I suggest some comments:

Introduction section:

What is the state of teenage pregnancy in Kenya? Or literature on teenage pregnancy?

Clarification is needed on what "definition" precisely is in this case?

Page 2: Line 58-59: I wonder if it is deliberate to position population divided and leave out other outcomes, especially equality.

Methods and materials

Page: 4: Line 90-92. Did you men interpretive analysis approach or interpretive focused ethnographic approach. Interpretive ethnographic approach if performed on ethnography enquiry- grounded on (participant) observation and inquiry.

Page 4: Line 103: on Theoretical underpinning. Why in methods and material section? Is this the structure of the journal?

Page 6: line 117. You mention purposive sampling: Provide clear sampling and recruitment of study participants.

Line 146-7: Who read through transcripts?

Page 8-9: Line 169: You have listed subjects organizations, did you get consent to include their organizations, and how does this play out in their confidentiality? A key stakeholder I expected to see here is the Ministry of Education, what is the reason for their exclusion? It would be interesting to find out how adolescent pregnancy is defined in education sector? Also excluded are donor organizations, any reason for this?

Findings section

Page 9-10: line 183-190: the excerpts provide different ways of delaying pregnancies: try to analyse and interpret the direct voice before moving to another section. Generally the excerpts are rich but under analysed.

Page 11: 225: Culture and religious: Unless you mean traditional religions? If not, culture and religion cannot define teenage pregnancy in the same way; those are two different views. In the list of subjects I have seen inter-religious representative does this subject represent the views of the cultural?

Page 12: line 231: I suggest you include, if any, some quote from the NGOs and the CSOs to qualify your etic interpretation.

Pages 18-21: from line 360: Discussion section: I was looking forward to this section, especially the intersections of arenas, and implications in furthering of understanding the dilemmas of teenage pregnancies in these arenas. In its current version, discussion is mainly a short summary and a number of propositions. You need to help readers see the broader significance of the work and how it relates to what is already known (or what we still need to understand) in this area.

I suggest you make a strong argument for the policy or recommend further studies.
