# Supplementary material for: Political prioritization and the competing definitions of adolescent pregnancy in Kenya: An application of the Public Arenas Model
Source: PLoS One. 2020 Sep 14;15(9):e0238136. doi: 10.1371/journal.pone.0238136 (PMC7489501; doi:10.1371/journal.pone.0238136)
Supplement: Attachment — Submitted filename: Response to reviewers.doc [file pone.0238136.s005.doc]

6 August. 2020

Thank you for your time and careful review of our manuscript “Political prioritization and the competing definitions of adolescent pregnancy in Kenya: An application of the Public Arenas Model.” We appreciate the feedback.

We have addressed your comment in the revised manuscript and have detailed our response to the query below (in blue font).

Thank you very much for your time and consideration of this revised manuscript for publication in PLOS ONE. If there is any additional information or details about the study that we can provide, please do not hesitate to contact me. We look forward to your response.

Sincerely,

Authors

Additional Editor Comments (if provided):

That no participants were drawn from the Ministry of Education is a key limitation to your study and this should be pointed out as such.

**Line 396-400:** Secondly, we did not interview a representative from the ministry of education, which is an important arena and stakeholder of adolescents. Although we interviewed a tertiary level education sector representative, we acknowledge that a large proportion of the adolescents will be in primary or high school and that this important opinion is missed in this paper.
